# Intraoperative Confocal Laser Endomicroscopy Detects Prostate Cancer at the Single-Cell Level with High Specificity and in Real Time: A Preclinical Proof of Concept

**DOI:** 10.3390/ph18060841

**Published:** 2025-06-04

**Authors:** Ann-Christin Eder, Jessica Matthias, Francois Lacombe, Lisa-Charlotte Domogalla, Antoine Jacques, Nils Steinacker, Gaetan Christien, Elodie Martin, Aline Criton, Matthias Eder

**Affiliations:** 1 Department of Nuclear Medicine, University Medical Center Freiburg, Faculty of Medicine, University of Freiburg, 79106 Freiburg, Germany; 2 Division of Radiopharmaceutical Development, German Cancer Consortium (DKTK), Partner Site Freiburg, Freiburg, Germany and German Cancer Research Center, 69120 Heidelberg, Germany; 3 Department of Optical Nanoscopy, Max Planck Institute for Medical Research, 69120 Heidelberg, Germany; 4 Mauna Kea Technologies, 75010 Paris, France

**Keywords:** prostate cancer, guided surgery, intraoperative microscopy, PSMA, confocal laser endomicroscopy

## Abstract

In prostate cancer (PCa) surgery, precise tumor margin identification remains challenging despite advances in surgical techniques. This study evaluates the combination of tumor-specific near-infrared imaging with the PSMA-targeting molecule PSMA-914 and optical endomicroscopy (NIR-pCLE) for single-cell-level tumor identification in a preclinical proof of concept. **Methods:** NIR-pCLE imaging of varying PSMA-914 concentrations was performed on PSMA-positive LNCaP and PSMA-negative PC-3 cells using Cellvizio^®^ 100 with pCLE Confocal Miniprobes™. To identify optimal PSMA-914 dosing for in vivo imaging, different doses (0–10 nmol) were evaluated using NIR-pCLE, Odyssey CLx imaging, and confocal microscopy in an LNCaP tumor-bearing xenograft model. A proof of concept mimicking a clinical workflow was performed using 5 nmol [^68^Ga]Ga-PSMA-914 in LNCaP and PC-3 tumor xenografts, including PET/MRI, in/ex vivo NIR-pCLE imaging, and microscopic/macroscopic imaging. **Results:** NIR-pCLE detected PSMA-specific fluorescence at concentrations above 30 nM in vitro. The optimal dose was identified as 5 nmol PSMA-914 for NIR-pCLE imaging with cellular resolution in LNCaP xenografts. PET/MRI confirmed high tumor uptake and a favorable distribution profile of PSMA-914. NIR-pCLE imaging enabled real-time, single-cell-level detection of PSMA-positive tissue, visualizing tumor heterogeneity, confirmed by ex vivo microscopy and imaging. **Conclusions:** This preclinical proof of concept demonstrates the potential of intraoperative PSMA-specific NIR-pCLE imaging to visualize tissue structures in real time at cellular resolution. Clinical implementation could provide surgeons with valuable additional information, potentially advancing PCa patient care through improved surgical precision.

## 1. Introduction

The introduction of cancer-targeting diagnostic radiopharmaceuticals has paved the way for the precise detection and localization of prostate cancer (PCa) by PET/CT imaging, providing valuable information for cancer staging and subsequent treatment planning [1,2]. A main therapy strategy in PCa care is the surgical removal of malignancies. However, current intraoperative technology does not provide real-time diagnostic information, limiting the accurate localization and delineation of tumor margins during surgery, and subsequent histopathologic analysis can take several days [3]. This increases the risk of tumor tissue being left behind and/or healthy tissue being removed instead. Several studies indicate that a positive surgical margin (PSM) is a risk factor for disease progression after surgery [4]. The incidences of biochemical recurrence (BCR) and local recurrence after surgery are independently correlated with PSMs [5,6,7].

Due to this clinical demand, recent developments have focused on the introduction of real-time cancer-specific in vivo optical imaging with a resolution down to the cellular level. In the case of PCa, the prostate-specific membrane antigen (PSMA) represents an ideal target structure for cancer-specific diagnostics. The development of PSMA-targeting peptidomimetics has already remarkably advanced the diagnostics and therapy of PCa, specifically highlighted by the theranostic agent PSMA-617 (approved by the US Food and Drug Administration (FDA) and European Medicines Agency (EMA) in 2022 as a last-line therapeutic for metastatic castration-resistant PCa (mCRPC)) [8,9]. Based on this low-molecular-weight peptidomimetics platform, the first PSMA-targeting hybrid molecules have been developed, comprising both a radioactive and fluorescent label to support pre- and intraoperative navigation [10,11,12,13]. The radiolabel enables preoperative, noninvasive imaging (e.g., PET/CT), and the fluorescent label subsequently provides intraoperative visual guidance. With the research field of targeted hybrid agents still in its infancy, the first clinical application of the PSMA-11-derived peptidomimetic PSMA-targeting hybrid molecule PSMA-914 demonstrated the potential of this technology for the surgical treatment of PCa patients [14]. While PSMA-914 allowed for macroscopic tumor detection, its potential to identify cancer at the cellular level is unknown.

Probe-based confocal laser endomicroscopy (pCLE) is an endoscopic and laparoscopic technique that promises cellular resolution during in situ tissue imaging. A preliminary study conducted with 21 patients scheduled for radical prostatectomy has convincingly demonstrated the intraoperative observation of microscopic features of prostatic and periprostatic tissues in vivo and ex vivo with the Cellvizio^®^ 100 Series combined with the CelioFlex™ Confocal Miniprobe™ [15]. Other recent studies have additionally highlighted the benefit of pCLE in the fluorescence-based real-time detection of living cells during bronchoscopy [16], visualization and characterization of hepatic lesions [17], and identification of cells from solitary pulmonary nodules [18,19].

The present study aims at evaluating the compatibility of PSMA-targeting hybrid molecules with pCLE for the real-time detection of PCa at the single-cell level during surgery. To this aim, the PSMA-914 fluorescence was detected with a thin, flexible pCLE Confocal Miniprobe™, combining the PCa specificity of the PSMA-targeting NIR tracer with the cellular resolution of pCLE (resulting method: NIR-pCLE). As a proof of concept, NIR-pCLE imaging of the hybrid molecule PSMA-914 was performed in vitro and in vivo on PSMA-expressing cells and tumor xenografts.

## 2. Results

### 2.1. Specific NIR-pCLE Detection of PSMA-Positive Cells In Vitro

In the first step, the feasibility of NIR-pCLE real-time detection of the PSMA-targeting hybrid molecule PSMA-914 was evaluated in vitro with pCLE using the Cellvizio^®^ system (Supplemental Figure S1). The fluorescence signal of PSMA-expressing LNCaP cells incubated with increasing concentrations of PSMA-914 (0–1000 nM) was measured using three different pCLE Confocal Miniprobes^TM^ (AlveoFlex^TM^-C, CholangioFlex^TM^-C^,^ and GastroFlex^TM^ UHD-C) (Supplemental Video S1). A PSMA-914-specific signal was detected with AlveoFlex^TM^-C and CholangioFlex^TM^-C for concentrations > 30 nM, and with GastroFlex^TM^ UHD-C for concentrations > 100 nM (Table 1). PSMA specificity was confirmed with PSMA-negative PC-3 cells, showing only a negligible fluorescence signal for all tested PSMA-914 concentrations.

### 2.2. Dose Finding for PSMA-Specific NIR-pCLE Imaging In Vivo

**Figure 1:**
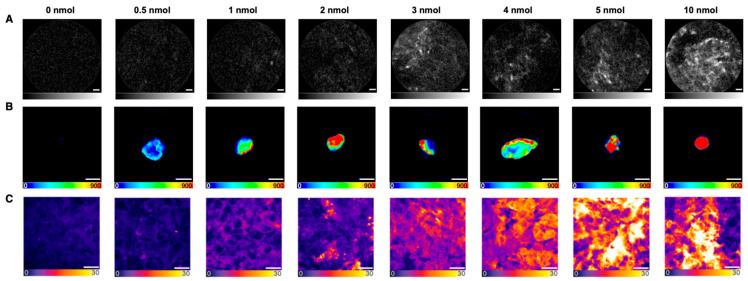
Fluorescence imaging of screened PSMA-914 doses in LNCaP tumor tissue 2 h p.i.. Representative images of (**A**) NIR-pCLE (in vivo, scale bar 20 μm, fluorescence intensity in arbitrary units, gray scale adjusted to min and max counts), (**B**) Odyssey CLx (ex vivo whole organs, scale bar 5 mm, raw fluorescence data are shown, color-coded by photon counts per pixel), and (**C**) confocal microscopy (ex vivo tissue cryosections, scale bar 20 μm, raw fluorescence data are shown, color-coded by photon counts per pixel) of 0/0.5/1/2/3/4/5/10 nmol PSMA-914 administered to LNCaP tumor-bearing mice (0 nmol (*n* = 3), 0.5/1/2/3/4/10 nmol (*n* = 1), 5 nmol (*n* = 2)) in the context of the dose finding study.

For study planning of the in vivo proof-of-concept study, the optimal PSMA-914 dose was identified by NIR-pCLE imaging in vivo and ex vivo 2 h after administering a range of doses (0.5/1/2/3/4/10 nmol (*n* = 1), 5 nmol (*n* = 2)) in a PSMA-positive xenograft mouse model. Tumor xenografts without drug injection served as reference (*n* = 3). NIR-pCLE imaging detected a strong fluorescence signal in PSMA-positive LNCaP tumors for doses >2 nmol, while the fluorescence signal in non-target organs (muscle, heart, spleen) and blood was negligible (Figure 1, Supplemental Figures S2 and S3, Supplemental Tables S1–S6). LNCaP tumors were visualized on a single-cell level, revealing clusters of PSMA-positive cells clearly delineated from surrounding non-fluorescent cells. The PSMA-914 fluorescence signal intensity saturated at 5 nmol with a non-significant increase at 10 nmol. The in vivo tumor-to-muscle ratio even decreased at 10 nmol due to tracer enrichment in non-target organs (Supplemental Table S5). Ex vivo NIR-pCLE imaging specifically confirmed the distinct fluorescence signal increase in the liver at 10 nmol PSMA-914 (Supplemental Table S6). Known to be excreted via the renal pathway, the PSMA-914 kidney signal was expectedly high with a dose-dependent increase for all mice (Supplemental Figure S3).

Subsequent Odyssey CLx imaging confirmed the correlation between increasing PSMA-914 doses and an increasing PSMA-914 fluorescence signal in the PSMA-positive tumor xenograft, while the references (LNCaP tumor xenografts without drug injection) consistently showed no significant fluorescence signal (Figure 1). However, non-target PSMA-914 localization was observed in non-target organs (liver, spleen, muscle) for doses of 2 nmol and higher (Supplemental Figure S4). Only negligible PSMA-914 uptake was detected in these organs for doses < 2 nmol, consistent with the previously published PSMA-914 tracer distribution (18).

**Figure 2:**
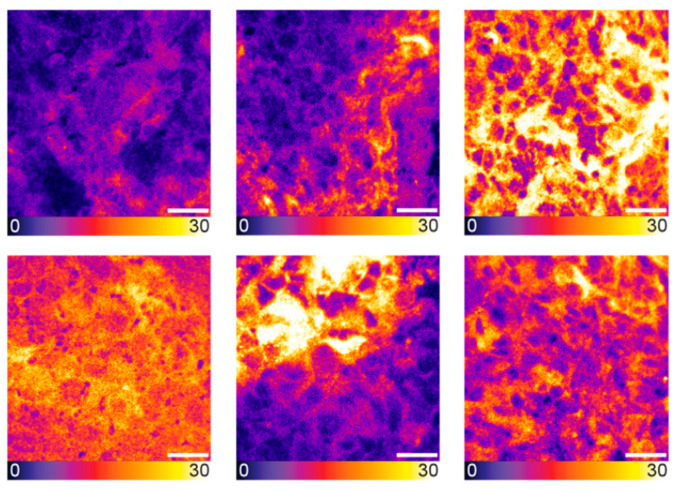
Heterogeneity in LNCaP tumor tissue as visualized by confocal microscopy. Exemplary confocal images of tumor tissue cryosections from LNCaP tumor-bearing mice, 2 h p.i. of 5 nmol PSMA-914 (*n* = 5). Scale bar 20 μm. Raw fluorescence data are shown, color-coded by photon counts per pixel.

After cryosectioning the tumor xenografts, confocal tissue imaging was performed to quantitatively compare the PSMA-914 signal strength between the different dose conditions based on the integrated fluorescence intensity. As the subcellular resolution of confocal microscopy revealed a heterogeneous tracer distribution in the cell population of the tumor tissue (Figure 2), the entire cryosections were explored, and a statistically significant number of fields of view (FOVs) were recorded to obtain a representative impression of each sample. As expected, the more PSMA-914 was administered, the more fluorescence signal was detected on average in the LNCaP tumor tissue (Figure 1, Supplemental Figures S5 and S6, Supplemental Tables S7–S11). Significant differences from the reference LNCaP xenografts without drug injection were reached with a PSMA-914 dose of ≥1 nmol. The integrated PSMA-914 fluorescence intensity peaked at a PSMA-914 dose of 4 nmol and did not significantly change at higher doses, indicating tracer saturation of the tumor tissue at ≤ 4 nmol and the absence of blocking effects up to doses of 10 nmol (Figure 1).

Taken together, providing sufficient fluorescence signal in NIR-pCLE-imaging and the lowest possible fluorescence signal in non-target organs, 5 nmol PSMA-914 was identified as the optimal dose for the following proof-of-concept study.

### 2.3. Detection of PSMA-914 Fluorescence at the Single-Cell Level: In Vivo Proof of Concept

**Figure 3:**
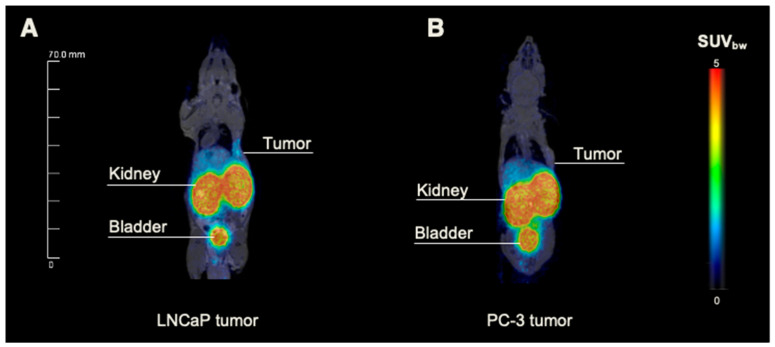
Small-animal PET/MRI of LNCaP and PC-3 tumor-bearing mice. Exemplary whole-body maximum intensity projections from small-animal PET/MR imaging of 5 nmol ^68^Ga-labeled PSMA-914 administered into the right flank of (**A**) LNCaP- and (**B**) PC-3-tumor-bearing BALB/c nu/nu mice 2 h p.i. (*n* = 3, respectively; corresponding SUV values in Supplemental Table S12).

**Figure 4:**
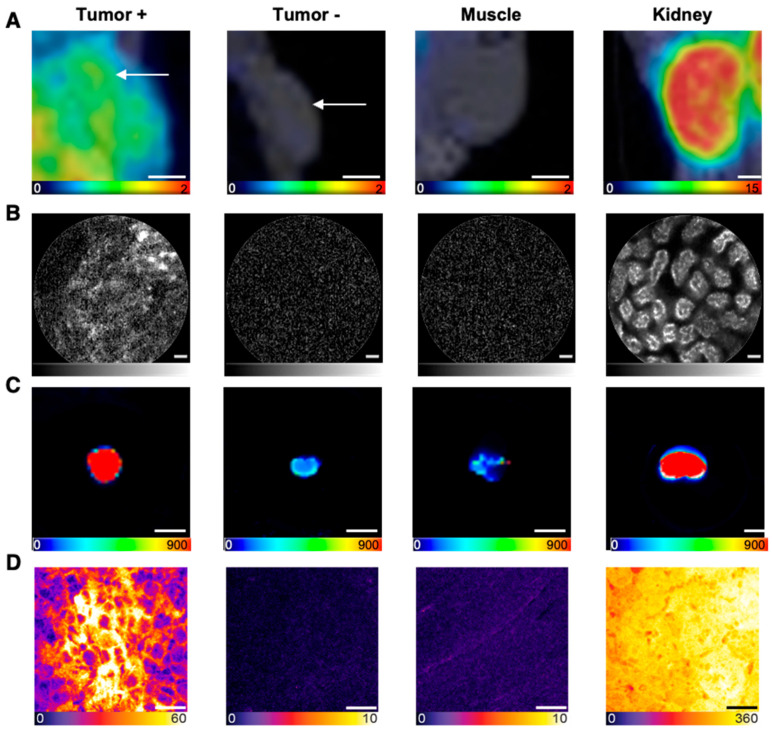
Multimodal imaging in the context of the in vivo proof-of-concept study. Representative images of (**A**) PET/MRI (coronal slices, in vivo, scale bar 2.5 mm, color scale in SUV values), (**B**) NIR-pCLE (in vivo, scale bar 20 μm, fluorescence intensity in arbitrary units, gray scale adjusted to min and max counts), (**C**) Odyssey CLx (ex vivo whole organs, scale bar 5 mm, raw fluorescence data are shown, color-coded by photon counts per pixel), and (**D**) confocal microscopy imaging (ex vivo tissue cryosections, scale bar 20 μm, raw fluorescence data are shown, color-coded by photon counts per pixel) of 5 nmol PSMA-914 in tumor, muscle, and kidney tissue of LNCaP and PC-3 xenograft-bearing mice, 2 h p.i. (*n* = 3, respectively).

The preclinical proof of concept was designed close to the clinical workflow (PET/MRI followed by optical imaging) to prepare the clinical combination of the technologies of PSMA-specific hybrid molecules and intraoperative imaging on the cellular level (NIR-pCLE). In PET/MRI, the previously reported tracer distribution of 0.5 nmol [^68^Ga]Ga-PSMA-914 was confirmed with 5 nmol of tracer in the PSMA-positive LNCaP xenograft mouse model (Figure 3 and Figure 4) [13]. [^68^Ga]Ga-PSMA-914 showed a tumor uptake of SUV_mean_ 0.29 ± 0.03 at 2 h p.i. and a clearance via the renal pathway (Figure 3, Supplemental Table S12). However, liver uptake (SUV_mean_ 0.42 ± 0.21) was also detected, consistent with the increased tracer uptake in non-target tissues at higher PSMA-914 doses in the dose finding study. Only negligible uptake of [^68^Ga]Ga-PSMA-914 was detected in non-target tissue (heart, lungs, muscle) and PSMA-negative PC-3 tumor xenografts (SUV_mean_ 0.06 ± 0.04), proving the PSMA-specificity of PSMA-914 (Figure 3, Supplemental Table S12).

**Figure 5:**
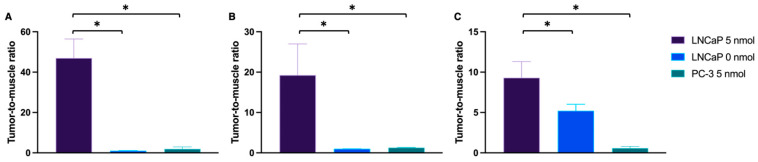
Tumor-to-muscle ratio of the fluorescence signal in NIR-pCLE and confocal microscopy. Tumor-to-muscle ratio of the median fluorescence signal (NIR-pCLE, in vivo) or the integrated fluorescence intensity (confocal microscopy, ex vivo tissue cryosections) detected in LNCaP and PC-3 tumor and muscle tissue, 2 h p.i. of 0 (*n* = 3) or 5 nmol PSMA-914 (*n* = 5, respectively) with (**A**,**B**) NIR-pCLE, using (**A**) CholangioFlex^TM^-C in vivo and (**B**) GastroFlex^TM^ UHD-C ex vivo, and (**C**) confocal microscopy in ex vivo cryosections. Means were compared using Student’s *t* test. *p*-Values < 0.05 (*) were considered statistically significant.

In line with the PET/MRI findings, in vivo and ex vivo NIR-pCLE imaging detected a significant tracer enrichment in LNCaP tumors (Figure 4, Supplemental Tables S5 and S6, Supplemental Video S2). PSMA-positive cancer cells in the tumor tissue were clearly visualized on a single-cell level at the µm scale. PC-3 tumors only showed a negligible fluorescence signal (Figure 4, Supplemental Figures S2 and S7, Supplemental Tables S13 and S14). Consequently, the tumor-to-muscle ratio of the median PSMA-914 fluorescence signal was significantly higher (*p* < 0.05) for LNCaP tissue than for PC-3 tumors (Figure 5, Supplemental Table S15). The fluorescence signal in non-target tissue was negligible, except for the kidneys, due to renal PSMA-914 excretion (Supplemental Table S6, Supplemental Videos 3–5). For each mouse, dose, and organ, a strong correlation between the in vivo and ex vivo NIR-pCLE data was found (Supplemental Figure S8).

The NIR-pCLE imaging results were confirmed with Odyssey CLx imaging, showing PSMA-specific tumor uptake of PSMA-914 accompanied by non-target uptake in liver and kidneys, marking the excretion route (Figure 4, Supplemental Figure S4). Other non-target organs only showed negligible PSMA-914 uptake.

In the last step, the sensitivity of confocal tissue imaging was exploited to challenge PSMA-914 specificity and to quantitatively investigate potential tracer enrichment at the target dose of 5 nmol in non-target tissue, specifically muscle tissue and PSMA-negative tumor tissue, based on the integrated PSMA-914 fluorescence intensity. The PSMA-positive tumor-to-muscle ratio was 9.3 ± 2.0 (*n*_FOV_ = 60) and the PSMA-negative tumor-to-muscle ratio was 0.6 ± 0.2 (*n*_FOV_ = 30) (Figure 4 and Figure 5, Supplemental Figure S6, Supplemental Tables S16 and S17), highlighting the tracer’s PSMA specificity. Qualitative investigation of the non-target accumulation of PSMA-914 over the range of tested doses revealed the absence of a significant tracer enrichment in muscle tissue (Supplemental Figure S9). As expected, confocal kidney tissue imaging could clearly visualize renal excretion in both PSMA-positive LNCaP tumor xenograft mice and in PSMA-negative PC-3 tumor xenograft mice (Supplemental Figure S9). Interestingly, the efficiency of renal clearance could only be qualitatively correlated with the dose (Supplemental Figure S10). It should be noted, however, that, for the doses ≠ 5 nmol, kidney tissue cryosections of only one animal each were measured, such that this observation can be explained by biological variability.

## 3. Discussion

Recent advances in oncological surgery allowed for the introduction of real-time diagnostics in the surgical theatre to improve surgical accuracy. Notably, cancer-specific fluorescence-based imaging offers real-time visualization and delineation of tumor tissue on the macroscopic level. Further advancing intraoperative fluorescence imaging to single-cell detection has the potential to provide a more precise tumor delineation by accurately identifying tumor margins in real time. To this end, tumor-targeted NIR confocal imaging and optical endomicroscopy have recently been combined in NIR-pCLE, and the new technology has been showcased, identifying cells from solitary nodules [18,19]. Applying this approach, the aim of the present study was to introduce NIR-pCLE for the specific detection of PCa at the cellular level. Exploiting PSMA-overexpression in PCa, the PSMA-targeting hybrid molecule PSMA-914, tagged with the NIR dye IRDye800CW, was detected with a thin, flexible pCLE Confocal Miniprobe^TM^ in this first preclinical proof of concept [13,14,18].

In the first step, three different pCLE Confocal Miniprobes^TM^ (AlveoFlex^TM^-C, CholangioFlex^TM^-C, GastroFlex^TM^ UHD-C) detected PSMA-specific fluorescence signal of PSMA-914 at low nanomolar concentrations on PSMA-positive LNCaP cells in vitro, using PSMA-negative PC-3 cells as the negative control for specificity. Subsequently, the optimal dose for preclinical detection in a BALB/c nu/nu LNCaP-bearing xenograft model was identified by screening doses of 0.5–10 nmol PSMA-914 with NIR-pCLE and confocal microscopy for fluorescence detection on the cellular level and Odyssey CLx imaging for fluorescence detection on the tissue level. NIR-pCLE visualized PSMA-positive cells in real time for doses ≥ 3 nmol PSMA-914 with a fluorescence signal saturating at 5 nmol both in vivo and ex vivo. Confocal microscopy confirmed this observation but detected significant differences from the reference LNCaP xenografts without drug injection, already for PSMA-914 doses of ≥1 nmol. Although real-time NIR-pCLE and static confocal microscopy are technically different, both imaging methods identified an optimal dose for reliable tumor tissue detection with cellular resolution in the range of 5 nmol PSMA-914, observed tracer saturation of the tumor tissue at the same dose, and did not find blocking effects up to PSMA-914 doses of 10 nmol.

Macroscopic Odyssey CLx imaging confirmed these findings but already detected PSMA-914 fluorescence signal for doses ≥ 0.5 nmol. In line with previous findings, non-target tissue was devoid of PSMA-914 fluorescence signal for doses < 2 nmol, except for kidneys as part of the excretion route [13]. For PSMA-914 doses of ≥2 nmol, a considerable signal increase was seen in liver, spleen, and muscle, most likely due to higher tracer concentrations in the blood resulting in increased non-specific background uptake in well-perfused organs, most pronounced at the 10 nmol dose. The distinct signal increase in the liver at 10 nmol PSMA-914 was also detected by NIR-pCLE. While macroscopic imaging reliably detected PSMA-914 in tumor and non-target tissue at the lowest tracer doses as compared to the other two imaging methods, it lacked cellular resolution and real-time output.

The three imaging methods were collectively indicative of a 5 nmol PSMA-914 dose as being optimal for reliable fluorescence detection in tumor tissue, with a negligible signal in non-target tissue. Consequently, this dose was used for the following preclinical proof of concept.

In the proof-of-concept study, [^68^Ga]Ga-PSMA-914-PET/MRI was followed by NIR-pCLE imaging with ex vivo confirmation of the intraoperative findings, mimicking a potential clinical workflow. PET/MRI confirmed the previously reported PSMA-specific tracer distribution of [^68^Ga]Ga-PSMA-914, showing high and PSMA-specific tumor uptake and fast renal excretion [13]. In line with the PET/MRI findings, subsequent in vivo NIR-pCLE imaging detected PSMA-positive tumor cells with high specificity, as confirmed by experiments without injection of PSMA-914 or with a PC-3-bearing PSMA-negative tumor xenograft. The high resolution visualized PSMA-positive cell clusters in the tumor tissue surrounded by non-fluorescent cells. A similar tumor heterogeneity was also observed by ex vivo confocal microscopy and is assigned to a variability in PSMA-expression levels and/or non-homogeneous tracer distribution in the tumor xenograft. Only a negligible PSMA-914 fluorescence signal was present in NIR-pCLE, confocal microscopy, and Odyssey CLx images of non-target organs (blood, lung, heart, muscle), providing a high contrast necessary for precise tumor cell identification with NIR-pCLE. For all three imaging methods, the highest PSMA-914 fluorescence signal was detected in kidney tissue due to the tracer’s renal excretion.

The strong correlation of in vivo and ex vivo NIR-pCLE data, consistent with the ex vivo findings of confocal microscopy and Odyssey CLx imaging, indicates a high photometric consistency of this approach despite the challenges of a different imaging access (direct transcutaneous access in vivo versus surgical tissue exposure ex vivo) or the use of different pCLE Miniprobe^TM^ probes. When compared to existing intraoperative technologies such as PSMA PET-CT of excised tissue or radioguided surgery with intraoperative probes, NIR-pCLE offers distinct advantages [20,21,22]. These include single-cell-level resolution (μm scale) with real-time visual feedback versus macroscopic information, immediate visualization versus delayed image processing, reduced radiation exposure during surgery, and access to challenging anatomical regions with the thin, flexible probe. This combination of features addresses significant limitations of current technologies, potentially enhancing surgical precision in the identification of tumor margins during PCa surgery.

Taken together, the successful implementation of this approach in a preclinical proof of concept encourages further in-depth translational studies to assess the clinical potential of the methodology. While preclinical dosing schemes are usually not directly translatable to the clinical setting, our findings indicate that a clinical application presumably requires a different dosing scheme for single-cell imaging than for macroscopic imaging. To achieve sufficient fluorescence detection at the cellular level, a higher dose of PSMA-914 was needed in this preclinical study. However, this assumption is only speculative and must be validated in a clinical study. While the relatively small size and softness of the mouse xenograft tumors presented challenges for handling and imaging with the pCLE-probes, similar limitations are not expected to be met in a clinical setting.

It is important to acknowledge that while LNCaP and PC-3 cell lines provided ideal PSMA-positive and PSMA-negative controls for this proof-of-concept study, they represent only a subset of the heterogeneous PSMA expression observed in clinical prostate cancer. The approach’s performance in detecting positive surgical margins will need to be evaluated in future clinical studies where the full spectrum of PSMA expression and the challenges of intraoperative imaging can be assessed.

Additionally, while our current study utilized single tumor xenografts, the clinical reality of prostate cancer often involves multifocal disease. The cellular-level resolution of NIR-pCLE could potentially address limitations of conventional imaging techniques like PSMA PET-CT, which may underestimate disease burden in multifocal settings. The flexibility of the pCLE Confocal Miniprobe™ enables examination of multiple suspicious areas during surgery. However, these potential advantages for multifocal disease detection remain speculative until validated in appropriate clinical settings.

With this perspective, combining pCLE for real-time detection at the single-cell level with a PSMA-targeting fluorescent agent is expected to pave the way for further advances in PCa management. Exploiting the overexpression of PSMA, this approach might visualize individual PCa cells during surgery, allowing for the precise delineation of surgical margins on the µm scale to improve surgical accuracy and outcome.

## 4. Materials and Methods

### 4.1. Study Drug and Radiolabeling

The study drug Glu-urea-Lys-(HE)_3_-HBED-CC-IRDye800CW (PSMA-914; IRDye800CW Absorption_max_ 774 nm, Emission_max_ 789 nm) was synthesized as described previously (Supplemental Figure S1) [13]. Details on radiolabeling are provided in the Supplementary Materials.

### 4.2. In Vitro Imaging

Details on cell culturing and seeding are provided in the Supplementary Materials. On the day of the experiment, RPMI medium was removed, and the cells were incubated with 250 µL of cold PSMA-914 dilution (10/30/60/100/250/500/1000 nM) for 1 h at 37 °C with 5% CO_2_. Subsequently, the RPMI medium was removed, and cells were washed once with 500 µL cold PBS. Finally, imaging was performed in 1 mL FluoroBrite medium. NIR-pCLE imaging was performed using the Cellvizio^®^ 100 series for visualization of cells and microstructures with an excitation wavelength of 785 nm and receiving fluorescence signals from 810 nm to 870 nm. Several pCLE Confocal Miniprobes^TM^ were used: AlveoFlex^TM^-C, CholangioFlex^TM^-C, and GastroFlex^TM^ UHD-C. Details on the NIR-pCLE device are provided in the Supplementary Materials.

### 4.3. In Vivo Imaging Study

For the experimental tumor models, 1 × 10^7^ cells of LNCaP (*n* = 14) or 5 × 10^6^ cells of PC-3 (*n* = 3) (in 50% Matrigel; Becton Dickinson) were subcutaneously inoculated into the flank of 7- to 8-week-old male BALB/c nu/nu mice (Janvier). For imaging, the ^68^Ga-labeled compound PSMA-914 was injected into a tail vein (^68^Ga-labeled 5 nmol PSMA-914 (LNCaP and PC-3 xenografts, *n* = 3, respectively) in 0.9% NaCl (pH 7). At 2 h after injection, mice were anesthetized (2% isoflurane) and PET/MRI was performed with a 3T PET/MRI scanner (Bruker) (details provided in the Supplementary Materials). After PET/MRI or 2 h p.i. of non-radioactive PSMA-914 (0 nmol (*n* = 3), 0.5/1/2/3/4/10 nmol (*n* = 1), 5 nmol (*n* = 2)) in the dose finding study, optical imaging of the subcutaneous tumor and organs of interest was subsequently performed with pCLE with the CholangioFlex^TM^-C probe. After in vivo imaging of the surface and the interior of the tumor and muscle tissue under constant isoflurane anesthesia, mice were sacrificed, and the tumor and organs of interest were dissected and imaged ex vivo with the GastroFlex^TM^ UHD-C probe (details provided in the Supplementary Materials, Supplemental Tables S1–S4).

Additionally, tumor and organs were imaged ex vivo using the Odyssey CLx system (LI-COR Biosciences, Bad Homburg vor der Höhe, Germany, excitation wavelength 800 nm). Finally, tumor and organs were embedded in TissueTek and directly frozen. Cryosections (10 µm) for confocal microscopy were afterwards prepared using a Leica Cryostat.

### 4.4. Confocal Microscopy

For confocal imaging, the cryosections were washed with PBS three times for 5 min and mounted in Mowiol. All confocal data were acquired with a custom-built STED (Stimulated Emission Depletion) system similar to the one published by Gorlitz et al. [23]. Details on the custom-built system and imaging are described in the Supplementary Materials.

### 4.5. Statistical Aspects

Experiments were performed at least in triplicate, except the dose finding study (*n* = 1). Quantitative data are expressed as mean ± SD. The numbers of replicates n are given in the respective figure or table captions, and further statistical aspects are provided in the Supplementary Materials.

## 5. Conclusions

This preclinical proof-of-concept study successfully demonstrated the feasibility of combining the PSMA-specific hybrid molecule PSMA-914 with NIR-pCLE imaging for the intraoperative fluorescence detection of PCa at the single-cell level in real time. The technology fusion allowed for the specific identification of PSMA-positive cells, visualizing heterogeneity in tumor tissue with a high image contrast to surrounding structures. It promises to improve the surgical care of PCa patients by providing advanced intraoperative real-time information to maximize surgical accuracy.

## Figures and Tables

**Table 1:** Quantification of NIR-pCLE imaging of PSMA-914 in vitro. Signal-to-noise ratio of different PSMA-914 concentrations (10/30/60/100/250/500/1000 nM) in vitro on PSMA-positive LNCaP cells or PSMA-negative PC-3 cells detected with the Cellvizio^®^ 100 series using pCLE Confocal Miniprobes™ (AlveoFlex^TM^-C, CholangioFlex^TM^-C, GastroFlex^TM^ UHD-C).

PSMA-914 [nM]	0	10	30	60	100	250	500	1000
LNCaP—AlveoFlex^TM^ -C	0.0	0.6	3.3	5.0	9.7	19.2	14.0	38.4
LNCaP—CholangioFlex^TM^-C	0.1	0.3	11.4	11.1	1.7	25.2	26.8	43.0
LNCaP—GastroFlex^TM^ UHD-C	0.0	0.2	0.0	0.0	3.3	85.9	119.0	268.0
PC-3—AlveoFlex^TM^ -C	0.0	0.1	0.0	0.0	0.0	0.0	0.0	0.0
PC-3—CholangioFlex^TM^-C	0.0	0.1	0.1	0.0	0.0	0.0	0.0	0.0
PC-3—GastroFlex^TM^ UHD-C	0.0	0.0	0.0	0.0	0.0	0.0	0.0	0.0

## Data Availability

Data are contained within the article and Supplementary Materials.

## Supplementary Materials

The following supporting information can be downloaded at https://www.mdpi.com/article/10.3390/ph18060841/s1, Supplemental Figure S1: Chemical structure of PSMA-914; Supplemental Figure S2: Quantification of in vivo NIR-pCLE imaging of different PSMA-914 doses with the miniprobe CholangioFlex^TM^-C in xenograft mice; Supplemental Figure S3: Quantification of ex vivo NIR-pCLE imaging of different PSMA-914 doses with the miniprobe GastroFlex^TM^ UHD-C in LNCaP tumor-bearing mice; Supplemental Figure S4: Odyssey CLx imaging of non-target organs 2 h p.i. after injection of 0–10 nmol PSMA-914; Supplemental Figure S5: Quantitative confocal image analysis of different PSMA-914 doses in LNCaP tumor and muscle tissue; Supplemental Figure S6: Tumor-to-muscle ratio of the fluorescence signal of different PSMA-914 doses in confocal microscopy of LNCaP and PC-3 tumor tissue; Supplemental Figure S7: Quantification of ex vivo NIR-pCLE imaging of 5 nmol PSMA-914 with the miniprobe GastroFlex^TM^ UHD-C in PC-3 tumor-bearing mice; Supplemental Figure S8: Correlation between the mean in vivo and ex vivo NIR-pCLE fluorescence signal in LNCaP tumor tissue; Supplemental Figure S9: Quantitative comparison of the signal strength of 5 nmol PSMA-914 in confocal imaging of LNCaP/PC-3 tumor, muscle, and kidney tissue; Supplemental Figure S10: Dose-dependent PSMA-914 fluorescence signal in kidney tissue as visualized by confocal microscopy; Supplemental Table S1: Number of frames collected ex vivo with GastroFlex^TM^ UHD-C in organs and tumor of LNCaP-bearing BALB/c nu/nu mice 2 h p.i. of PSMA-914; Supplemental Table S2: Number of frames collected in vivo with CholangioFlex^TM^-C in muscle and tumor of LNCaP-bearing BALB/c nu/nu mice 2 h p.i. of PSMA-914; Supplemental Table S3: Number of frames collected ex vivo with GastroFlex^TM^ UHD-C and in vivo with CholangioFlex^TM^-C in organs and tumor of PC-3-bearing BALB/c nu/nu mice 2 h p.i. of PSMA-914; Supplemental Table S4: Total number of frames collected ex vivo with GastroFlex^TM^ UHD-C and in vivo with CholangioFlex^TM^-C in organs and tumors of LNCaP- and PC-3-bearing BALB/c nu/nu mice 2 h p.i. of PSMA-914; Supplemental Table S5: Tumor-to-muscle ratio of the median in vivo pCLE fluorescence signal detected in tumor and muscle tissue of LNCaP-bearing BALB/c nu/nu mice 2 h p.i. of PSMA-914. Probe: CholangioFlex^TM^-C; Supplemental Table S6: Organ-to-muscle ratio of the median ex vivo pCLE fluorescence signal detected in organ and muscle tissue of LNCaP-bearing BALB/c nu/nu mice 2 h p.i. of PSMA-914. Probe: GastroFlex^TM^ UHD-C; Supplemental Table S7: Integrated fluorescence intensity per area as detected with confocal imaging of different PSMA-914 doses in tumor and organ tissue cryosections of LNCaP-bearing BALB/c nu/nu mice 2 h p.i.; Supplemental Table S8: *p*-Values of the quantitative confocal image analysis of different doses of PSMA-914 in tumor tissue cryosections of LNCaP tumor-bearing BALB/c nu/nu mice 2 h p.i.; Supplemental Table S9: *p*-Values of the quantitative confocal image analysis of different doses of PSMA-914 in muscle tissue cryosections of LNCaP tumor-bearing BALB/c nu/nu mice 2 h p.i.; Supplemental Table S10: Tumor-to-muscle ratios of the mean integrated fluorescence intensity per area of confocal imaging of different PSMA-914 doses in tumor and muscle tissue cryosections of LNCaP- and PC-3-bearing BALB/c nu/nu mice 2 h p.i.; Supplemental Table S11: *p*-Values of the quantitative confocal image analysis of 5 nmol PSMA-914 in tumor, muscle, and kidney tissue cryosections of LNCaP- and PC-3 tumor-bearing BALB/c nu/nu mice 2 h p.i.; Supplemental Table S12: Mean SUV values of PET/MR imaging of 5 nmol ^68^Ga-labeled PSMA-914 in LNCaP- and PC-3 tumor-bearing BALB/c nu/nu mice 2 h p.i.; Supplemental Table S13: Tumor-to-muscle ratio of the median in vivo pCLE fluorescence signal detected in tumor and muscle tissue of PC-3-bearing BALB/c nu/nu mice 2 h p.i. of PSMA-914; Supplemental Table S14: Organ-to-muscle ratio of the median ex vivo pCLE fluorescence signal detected in organ and muscle tissue of PC-3-bearing BALB/c nu/nu mice 2 h p.i. of PSMA-914; Supplemental Table S15: Tumor-to-muscle ratio of the median ex vivo pCLE fluorescence signal in tumor and in muscle tissue of LNCaP- and PC-3-bearing BALB/c nu/nu mice 2 h p.i. of 0 or 5 nmol PSMA-914; Supplemental Table S16: Integrated fluorescence intensity per area of confocal imaging of 5 nmol PSMA-914 in organ cryosections of PC-3-bearing BALB/c nu/nu mice 2 h p.i.; Supplemental Table S17: *p*-Values of quantitative confocal image analysis of 5 nmol PSMA-914 in PC-3 tumor-bearing BALB/c nu/nu mice 2 h p.i.; Video S1: In vitro LNCaP cells—GFX; Video S2: Ex vivo LNCaP xenograft—GFX-5 nmol PSMA-914; Video S3: Ex vivo LNCaP xenograft—kidney—GFX-5 nmol PSMA-914; Video S4: Ex vivo LNCaP—muscle—GFX-5 nmol PSMA-914; Video S5: Ex vivo PC-3 xenograft—GFX-5 nmol PSMA-914.

## Abbreviations

The following abbreviations are used in this manuscript:

BCR	Biochemical recurrence
CT	Computed tomography
EMA	European Medicines Agency
FDA	US Food and Drug Administration
mCRPC	Metastatic castration-resistant prostate cancer
MRI	Magnetic resonance imaging
NIR	Near-infrared
PCa	Prostate cancer
PET	Positron emission tomography
pCLE	Probe-based confocal laser endomicroscopy
PSM	Positive surgical margin
PSMA	Prostate-specific membrane antigen

## References

[1] Schwarzenboeck S.M., Rauscher I., Bluemel C., Fendler W.P., Rowe S.P., Pomper M.G., Afshar-Oromieh A., Herrmann K., Eiber M. (2017). PSMA Ligands for PET Imaging of Prostate Cancer. J. Nucl. Med..

[2] Roberts M.J., Maurer T., Perera M., Eiber M., Hope T.A., Ost P., Siva S., Hofman M.S., Murphy D.G., Emmett L. (2023). Using PSMA imaging for prognostication in localized and advanced prostate cancer. Nat. Rev. Urol..

[3] Fernandes S., Williams G., Williams E., Ehrlich K., Stone J., Finlayson N., Bradley M., Thomson R.R., Akram A.R., Dhaliwal K. (2021). Solitary pulmonary nodule imaging approaches and the role of optical fibre-based technologies. Eur. Respir. J..

[4] Mauermann J., Fradet V., Lacombe L., Dujardin T., Tiguert R., Tetu B., Fradet Y. (2013). The impact of solitary and multiple positive surgical margins on hard clinical end points in 1712 adjuvant treatment-naive pT2-4 N0 radical prostatectomy patients. Eur. Urol..

[5] Boorjian S.A., Karnes R.J., Crispen P.L., Carlson R.E., Rangel L.J., Bergstralh E.J., Blute M.L. (2010). The impact of positive surgical margins on mortality following radical prostatectomy during the prostate specific antigen era. J. Urol..

[6] Novara G., Ficarra V., Mocellin S., Ahlering T.E., Carroll P.R., Graefen M., Guazzoni G., Menon M., Patel V.R., Shariat S.F. (2012). Systematic review and meta-analysis of studies reporting oncologic outcome after robot-assisted radical prostatectomy. Eur. Urol..

[7] Zorn K.C., Gallina A., Hutterer G.C., Walz J., Shalhav A.L., Zagaja G.P., Valiquette L., Gofrit O.N., Orvieto M.A., Taxy J.B. (2007). External validation of a nomogram for prediction of side-specific extracapsular extension at robotic radical prostatectomy. J. Endourol..

[8] Sartor O., de Bono J., Chi K.N., Fizazi K., Herrmann K., Rahbar K., Tagawa S.T., Nordquist L.T., Vaishampayan N., El-Haddad G. (2021). Lutetium-177-PSMA-617 for Metastatic Castration-Resistant Prostate Cancer. N. Engl. J. Med..

[9] Hofman M.S., Emmett L., Sandhu S., Iravani A., Joshua A.M., Goh J.C., Pattison D.A., Tan T.H., Kirkwood I.D., Ng S. (2021). [(177)Lu]Lu-PSMA-617 versus cabazitaxel in patients with metastatic castration-resistant prostate cancer (TheraP): A randomised, open-label, phase 2 trial. Lancet.

[10] Banerjee S.R., Pullambhatla M., Byun Y., Nimmagadda S., Foss C.A., Green G., Fox J.J., Lupold S.E., Mease R.C., Pomper M.G. (2011). Sequential SPECT and optical imaging of experimental models of prostate cancer with a dual modality inhibitor of the prostate-specific membrane antigen. Angew. Chem. Int. Ed. Engl..

[11] Schottelius M., Wurzer A., Wissmiller K., Beck R., Koch M., Gorpas D., Notni J., Buckle T., van Oosterom M.N., Steiger K. (2019). Synthesis and Preclinical Characterization of the PSMA-Targeted Hybrid Tracer PSMA-I&F for Nuclear and Fluorescence Imaging of Prostate Cancer. J. Nucl. Med..

[12] Baranski A.C., Schafer M., Bauder-Wust U., Roscher M., Schmidt J., Stenau E., Simpfendorfer T., Teber D., Maier-Hein L., Hadaschik B. (2018). PSMA-11-Derived Dual-Labeled PSMA Inhibitors for Preoperative PET Imaging and Precise Fluorescence-Guided Surgery of Prostate Cancer. J. Nucl. Med..

[13] Eder A.C., Schafer M., Schmidt J., Bauder-Wust U., Roscher M., Leotta K., Haberkorn U., Kopka K., Eder M. (2021). Rational Linker Design to Accelerate Excretion and Reduce Background Uptake of Peptidomimetic PSMA-Targeting Hybrid Molecules. J. Nucl. Med..

[14] Eder A.C., Omrane M.A., Stadlbauer S., Roscher M., Khoder W.Y., Gratzke C., Kopka K., Eder M., Meyer P.T., Jilg C.A. (2021). The PSMA-11-derived hybrid molecule PSMA-914 specifically identifies prostate cancer by preoperative PET/CT and intraoperative fluorescence imaging. Eur. J. Nucl. Med. Mol. Imaging.

[15] Lopez A., Zlatev D.V., Mach K.E., Bui D., Liu J.J., Rouse R.V., Harris T., Leppert J.T., Liao J.C. (2016). Intraoperative Optical Biopsy during Robotic Assisted Radical Prostatectomy Using Confocal Endomicroscopy. J. Urol..

[16] Wijmans L., Yared J., de Bruin D.M., Meijer S.L., Baas P., Bonta P.I., Annema J.T. (2019). Needle-based confocal laser endomicroscopy for real-time diagnosing and staging of lung cancer. Eur. Respir. J..

[17] Pierangelo A., Fuks D., Validire P., Benali A., Gayet B. (2017). Diagnostic accuracy of confocal laser endomicroscopy for the characterization of liver nodules. Eur. J. Gastroenterol. Hepatol..

[18] Kennedy G.T., Azari F.S., Bernstein E., Nadeem B., Chang A., Segil A., Carlin S., Sullivan N.T., Encarnado E., Desphande C. (2022). Targeted detection of cancer at the cellular level during biopsy by near-infrared confocal laser endomicroscopy. Nat. Commun..

[19] Kennedy G.T., Azari F.S., Bernstein E., Nadeem B., Chang A., Segil A., Sullivan N., Encarnado E., Desphande C., Kucharczuk J.C. (2022). Targeted detection of cancer cells during biopsy allows real-time diagnosis of pulmonary nodules. Eur. J. Nucl. Med. Mol. Imaging.

[20] Darr C., Costa P.F., Kahl T., Moraitis A., Engel J., Al-Nader M., Reis H., Kollermann J., Kesch C., Krafft U. (2023). Intraoperative Molecular Positron Emission Tomography Imaging for Intraoperative Assessment of Radical Prostatectomy Specimens. Eur. Urol. Open Sci..

[21] Schilham M.G.M., Somford D.M., Kusters-Vandevelde H.V.N., Hermsen R., van Basten J.P.A., Hoekstra R.J., Scheenen T.W.J., Gotthardt M., Sedelaar J.P.M., Rijpkema M. (2024). Prostate-Specific Membrane Antigen-Targeted Radioguided Pelvic Lymph Node Dissection in Newly Diagnosed Prostate Cancer Patients with a Suspicion of Locoregional Lymph Node Metastases: The DETECT Trial. J. Nucl. Med..

[22] Berrens A.C., Scheltema M., Maurer T., Hermann K., Hamdy F.C., Knipper S., Dell’Oglio P., Mazzone E., de Barros H.A., Sorger J.M. (2024). Delphi consensus project on prostate-specific membrane antigen (PSMA)-targeted surgery-outcomes from an international multidisciplinary panel. Eur. J. Nucl. Med. Mol. Imaging.

[23] Gorlitz F.H.P., Falk H.J., Kastrup L., Engelhardt J., Hell S.W. (2014). A STED Microscope Designed for Routine Biomedical Applications. Prog. Electromagn. Res..

