# Supplementary material for: Intraoperative Confocal Laser Endomicroscopy Detects Prostate Cancer at the Single-Cell Level with High Specificity and in Real Time: A Preclinical Proof of Concept
Source: Pharmaceuticals (Basel). 2025 Jun 4;18(6):841. doi: 10.3390/ph18060841 (PMC12196187; doi:10.3390/ph18060841)
Supplement: Supplementary file 1 [file pharmaceuticals-18-00841-s001.zip › pharmaceuticals-3529455-supplementary.pdf]

# Supplemental Information

## Intraoperative Confocal Laser Endomicroscopy Detects Prostate Cancer at the Single-Cell Level with High Specificity and in Real Time: A Preclinical Proof of Concept

Ann-Christin Eder[1,2*], Jessica Matthias[3#], Francois Lacombe[4], Lisa-Charlotte Domogalla[1,2], Antoine Jacques[4], Nils Steinacker[1,2], Gaetan Christien[4], Elodie Martin[4], Aline Criton[4], Matthias Eder[1,2]

[1] Department of Nuclear Medicine, University Medical Center Freiburg, Faculty of Medicine, University of Freiburg, Freiburg, Germany
[2] Division of Radiopharmaceutical Development, German Cancer Consortium (DKTK), partner site Freiburg, Freiburg, Germany and German Cancer Research Center, Heidelberg, Germany
[3] Department of Optical Nanoscopy, Max Planck Institute for Medical Research, Heidelberg, Germany
[#] present address: Abberior Instruments America, Bethesda, USA
[4] Mauna Kea Technologies, Paris, France
*Correspondence: ann-christin.eder@uniklinik-freiburg.de; Tel.: +49 761 270 74221

# Supplemental Methods

### Radiolabeling

For radiolabeling, the precursor peptide [15 nmol PSMA-914 in 2-[4-(2-hydroxyethyl)piperazin-1-yl]ethanesulfonic acid (HEPES) buffer (2.1 M, pH 4) with 0.5 µL ascorbic acid (0.1 mg/mL), 40 µL] was added to 40 µL $^{68}Ga^{3+}$ eluate (~40-60 MBq). The pH was adjusted to 4 using 30% NaOH and 10% NaOH. The reaction was performed at 95°C for 30 min. The radiochemical yield was analyzed by reversed-phase high performance liquid chromatography (RP-HPLC, Agilent, Column, Gradient) and reversed-phase thin layer chromatography (RP-TLC, 60 RP-18 F254S) with DMF/sodium acetate/HCl (pH = 4.5, 1:1) as the mobile phase.

### Cell culture and preparation of *in vitro* experiments

Cells (PSMA-positive: LNCaP cells (CRL-1740; ATCC), PSMA-negative: PC-3 cells (CRL-1435; ATCC)) were cultured in RPMI medium supplemented with 10% fetal calf serum, 1% penicillin/streptomycine and 1% sodium pyruvate (all from PAA), and grown at 37°C in humidified air with 5% $CO_2$. Trypsin-ethylenediaminetetraacetic acid (trypsin-EDTA; 0.25% trypsin, 0.02% EDTA, Invitrogen) was used to harvest the cells. Cell line authentication is

regularly performed and the authentication of the LNCaP and PC-3 cell lines was confirmed on 03/2023. For the *in vitro* imaging, cells were seeded (200.000/well LNCaP, 100.000/well PC-3) on poly-L-lysine (0.1 % in $H_2O$) pre-coated LabTek Chamber slides (Lab-Tek II, CC2, 4 chambers) and incubated for 24 h at 37°C with 5 % $CO_2$ in supplemented RPMI medium.

**PET/MR Imaging**

A non-triggered localizer and a T1-weighted 3D scan was applied for MR imaging followed by recording a static PET scan (20 min). Image reconstruction was done with the ParaVision software (OSEM 0.25 mm algorithm, 1 iteration) and data analysis was conducted in PMOD (version 3.7 and 4.3) with data converted to SUV images.

**NIR-pCLE device**

Three flexible, NIR-pCLE Confocal Miniprobes™ were used in conjunction with the Cellvizio® 100 Series F800 (Mauna Kea Technologies, Paris, France): AlveoFlex™-C, GastroFlex™ UHD-C and CholangioFlex™-C, with outer diameters of 1.4 mm, 2.6 mm and 0.85 mm, respectively. These miniprobes are all based on the same technology and materials, namely a bundle of optical fibers enclosed in a protective sheath, terminated with a miniaturized objective lens. The number of fibers in the bundle ranged from 10.000 in the CholangioFlex™-C (working distance of 55 µm, lateral resolution of 3.5 µm, maximum field of view of 325 µm) to 30.000 for the AlveoFlex™-C (working distance of >0µm, lateral resolution of 3.5 µm, maximum field of view of 600 µm) and for the GastroFlex™ UHD-C (working distance of 60 µm, lateral resolution of 1 µm, maximum field of view of 240 µm).

The Confocal Miniprobes™ were connected to the Cellvizio® 100 Series F800 system, a commercially available device with a 785 nm excitation laser, which is focused by design at the position of the entrance plane of the bundle of optical fibers, scanning the bundle's entire surface. The laser light was transmitted by each optical fiber sequentially and focused onto the tissue by the distal optical head of the Confocal Miniprobe™. Fluorescence light emitted by the tissue was collected with the Confocal Miniprobe™ and transmitted to the Cellvizio® system.

The fluorescence signal was de-scanned, optically filtered (detection window between 810 and 870 nm), digitalized, and processed on the fly by the Cellvizio® software to be displayed in real time as images on the display screen attached to the device.

The Cellvizio® 100 Series F800 allowed recordings 8.8 frames per second with the GastroFlex™ UHD-C or the AlveoFlex™-C miniprobe, and 12.3 frames per second with the CholangioFlex™-C miniprobes.

***Ex vivo* NIR-pCLE imaging**

For *ex vivo* imaging, pCLE was performed with the GastroFlex™ UHD-C probe on the surface of the lungs, inside and outside of the heart, liver, kidney, muscle, tumor and in blood. The recordings of the imaging sequence was 30-90 seconds, starting when the probe was in contact with the tissue (Supplemental Tables 1-4). To avoid the photobleaching phenomenon recordings were performed with the probe in movement. Three mice had to be excluded for the quantitative pCLE image analysis due to fluorescence contaminated pCLE-probes in two cases and muscle tissue in one case. Further details on quantification in NIR-pCLE sequences are described in the following section.

**Quantitative NIR-pCLE image analysis**

*Online subtraction of a 2D photometric zero*

The Cellvizio® system collected PSMA-914 fluorescence emitted by the few cells present in the confocal volume of each fiber of the miniprobe. Scaling the cells' amount of bound and internalized PSMA-914, the fluorescence signal can be faint and close to the noise level of the system, mostly because of the conditions during real-time and *in situ* imaging.

Therefore, the estimation of a robust photometric zero, i.e. the detected signal in total absence of PSMA-914 fluorescence, is of prime importance. This background is a combination of the tissue autofluorescence and the Raman scattering inside the individual fibers of the miniprobes.

The Cellvizio® system is equipped with an acquisition software which evaluates this photometric zero each time a miniprobe is connected to the system, by recording and averaging 60 images while the miniprobe is not yet in contact with tissue (i.e. air interface).

The photometric zero, individually assessed for each fiber, is a 2D map which can be systematically subtracted from the data during the actual measurements.

*pCLE data analysis*

The mean fluorescence of each NIR-pCLE imaging sequence was calculated using a dedicated MATLAB routine (R2021b, MathWorks), wich finds clusters in histograms allowing for the segmentation in NIR-pCLE frames. The mean fluorescence signal of each NIR-pCLE image sequence of the tumor was normalized on the mean fluorescence signal of each NIR-pCLE image sequence of the normal tissue from the same subject by dividing the mean tumor fluorescence signal by the background signal.

*Offline quantification of the recorded data*

For each mouse and each organ, each cell line, *in vitro*, *in vivo*, or *ex vivo*, the mean signal in each NIR-pCLE frame was calculated after a global photometric reference image (or photometric zero) was subtracted. From the collection of all such frames, the median value was extracted. The median was preferred to the mean, for this second step, to account for frequent outliers when using *in situ* endomicroscopy (e.g. miniprobe not in full contact with tissue, photobleaching artifacts).

**Confocal Microscopy**

Confocal imaging was performed on a custom-built STED system similar to the one published by Gorlitz et al. (1). The nanosecond pulsed 775 nm fiber laser (MPBC, usually employed as depletion laser for STED imaging) was used at 2.4 mW for excitation of PSMA-914, and a pulsed 470 nm diode laser (LDH-D-C470, PicoQuant) was used at 700 µW for excitation of tissue autofluorescence. Laser powers were measured in front of the microscope body. PSMA-

914 fluorescence was detected at 835/70 nm and tissue autofluorescence was detected at 525/50 nm. Dual color confocal images of muscle and tumor tissue were acquired in line multiplexing mode with a pixel size of 80 nm and a dwell time of 30 µs without line accumulation. To account for renal excretion of PSMA-914 and thus renal accumulation, dual color confocal images of kidney tissue were acquired in line multiplexing mode with a pixel size of 50 nm and a dwell time of 5 µs without line accumulation for the PSMA-914 channel, but with a line accumulation of 6 for the autofluorescence channel.

The integrated fluorescence intensity was extracted with a home-written ImageJ routine (*20*). This script creates a mask for each autofluorescence image based on a fixed signal intensity threshold distinguishing between tissue absence (set to “not-a-number”) and tissue presence (set to “1)”. Multiplying this mask with the corresponding raw PSMA-914 image generates an output image with which the integrated PSMA-914 fluorescence intensity can be evaluated exclusively in the relevant parts of the raw PSMA-914 image without summing up background from tissue-free sample regions.

**Statistical Aspects**

Bar plots depict mean ± SD of the measurements for replicate experiments. Violin plots present the average *ex vivo* signal per pixel over all recorded frames with a central line indicating the median. Box plots indicate the interquartile range (box), the median (center line) and whiskers down to the minimum and up to the maximum value, with each individual value as a point superimposed on the graph. If applicable, means were compared using Student´s *t* test (GraphPad Prism Version 8, GraphPad Software, Inc.). p-values < 0.05 were considered statistically significant.

## Supplemental Figures

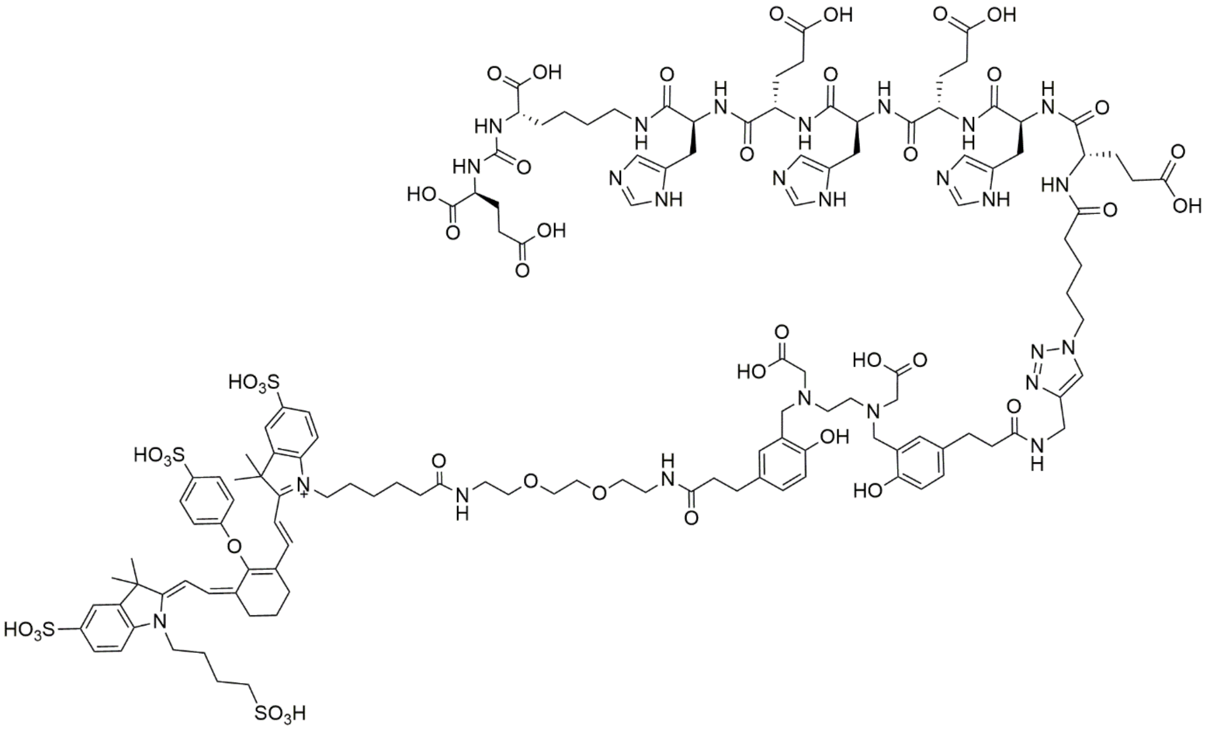

**Supplemental Figure S1. Chemical structure of PSMA-914.**

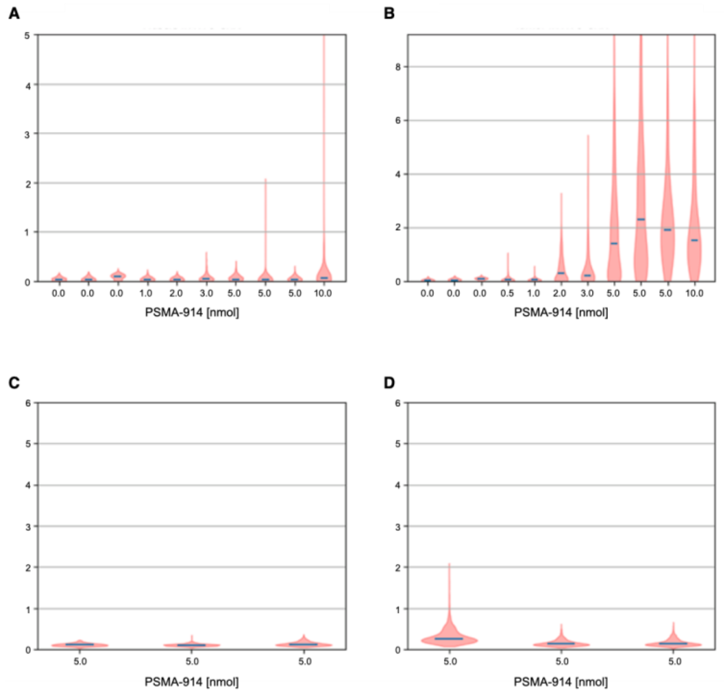


**Supplemental Figure S2. Quantification of *in vivo* NIR-pCLE imaging of different PSMA-914 doses with the miniprobe CholangioFlex$^{TM}$-C in xenograft mice.** Violin plots present the average *in vivo* signal per pixel, in Analog-to-Digital Units (ADUs), over all recorded frames with a central line indicating the median in (**A**) muscle (LNCaP xenograft), (**B**) tumor (LNCaP xenograft), (**C**) muscle (PC-3 xenograft) and (**D**) tumor (PC-3 xenograft). Information on the sample size (*n*) is given in the Supplemental Tables 2 and 3.

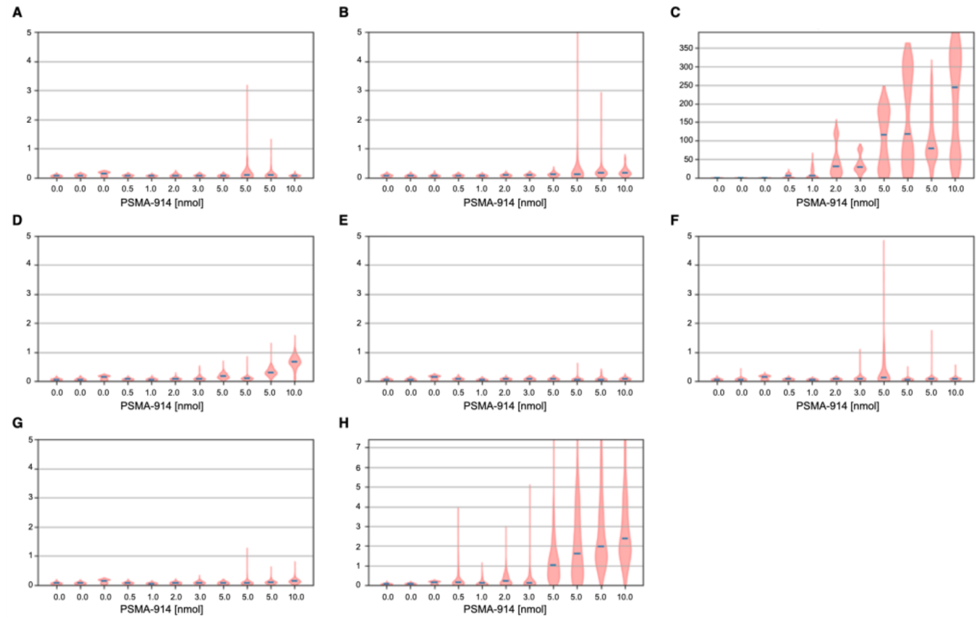


**Supplemental Figure S3. Quantification of *ex vivo* NIR-pCLE imaging of different PSMA-914 doses with the miniprobe GastroFlex$^{TM}$ UHD-C in LNCaP tumor-bearing mice.** Violin plots present the average *ex vivo* signal per pixel, in Analog-to-Digital Units (ADUs), over all recorded frames with a central line indicating the median in (**A**) blood, (**B**) heart, (**C**) kidney, (**D**) liver, (**E**) lung, (**F**) muscle, (**G**) spleen, (**H**) tumor. Information on the sample size (*n*) is given in the Supplemental Tables 2 and 3.

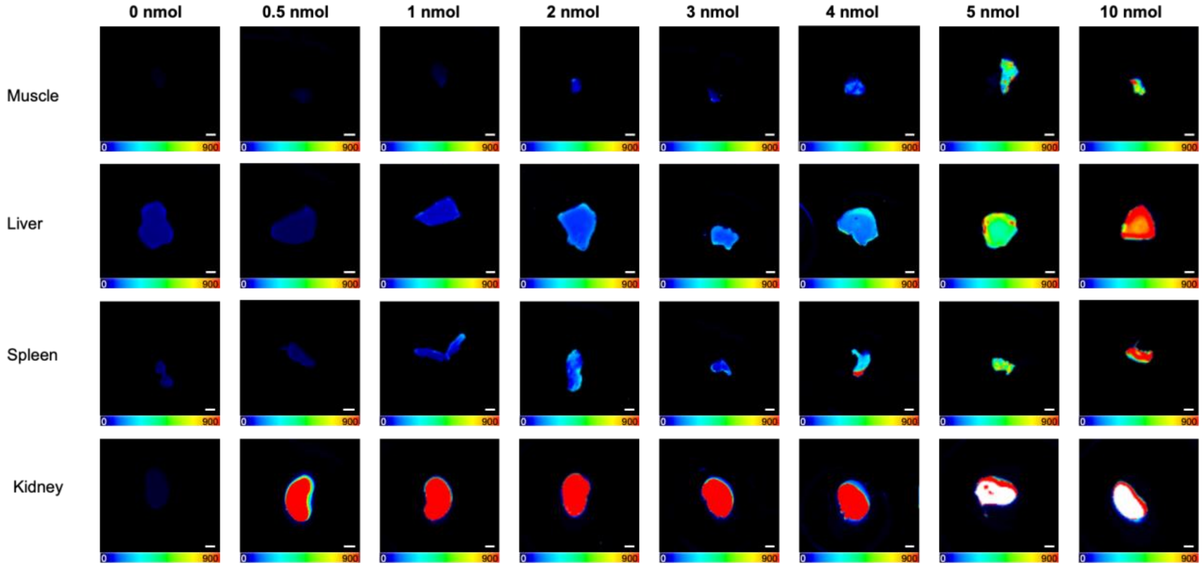


**Supplemental Figure S4. Odyssey CLx imaging of non-target organs 2 h p.i. after injection of 0 - 10 nmol PSMA-914.** Non-target organs of interest (muscle, liver, spleen, kidney) were imaged 2 h p.i. after injection of different PSMA-914 concentrations (0 - 10 nmol) with an excitation wavelength of 800 nm (scale bar: 5 mm, raw fluorescence data are shown, color-coded by photon counts per pixel).

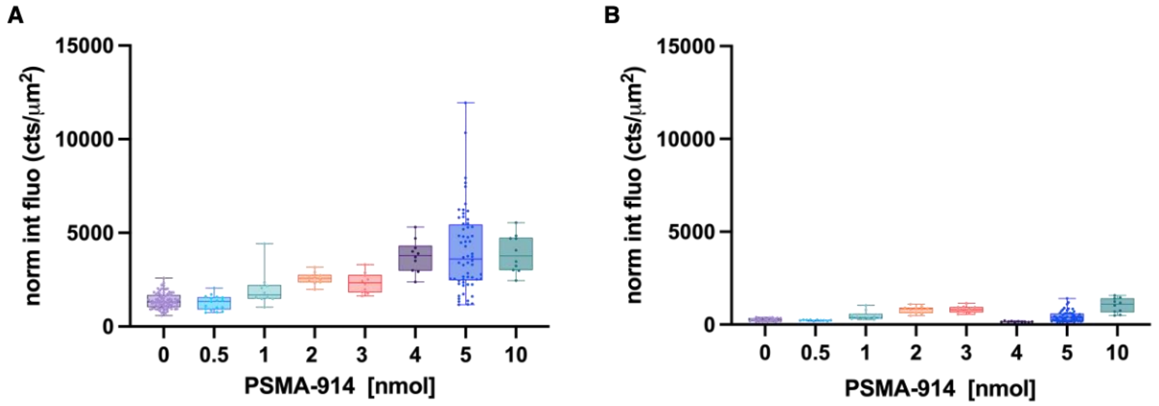


**Supplemental Figure S5. Quantitative confocal image analysis of different PSMA-914 doses in LNCaP tumor and muscle tissue.** Box plots present the integrated fluorescence intensity per area in confocal images acquired *ex vivo* with tumor (**A**) and muscle (**B**) tissue cryosections of LNCaP xenograft mice 2 h p.i. of 0/0.5/1/2/3/4/5/10 nmol PSMA-914, with a central line indicating the median. Information on the sample size (*n*) is given in the Supplemental Table 7. Information on significance testing (p-values) is given in Supplemental Tables 8 and 9.

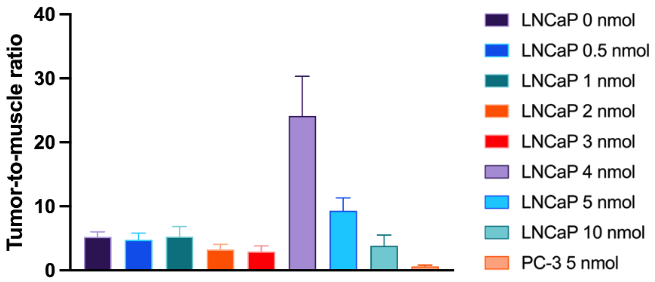


**Supplemental Figure S6. Tumor-to-muscle ratio of the fluorescence signal of different PSMA-914 doses in confocal microscopy of LNCaP- and PC-3-tumor tissue.** Tumor-to-muscle ratio of the integrated fluorescence intensity per area of confocal images acquired *ex vivo* with tumor tissue cryosections of LNCaP- and PC-3 xenograft mice 2 h p.i. of 0/0.5/1/2/3/4/5/10 nmol PSMA-914. Data are plotted as mean ± SD. Information on the sample size (*n*) is given in the Supplemental Table 7.

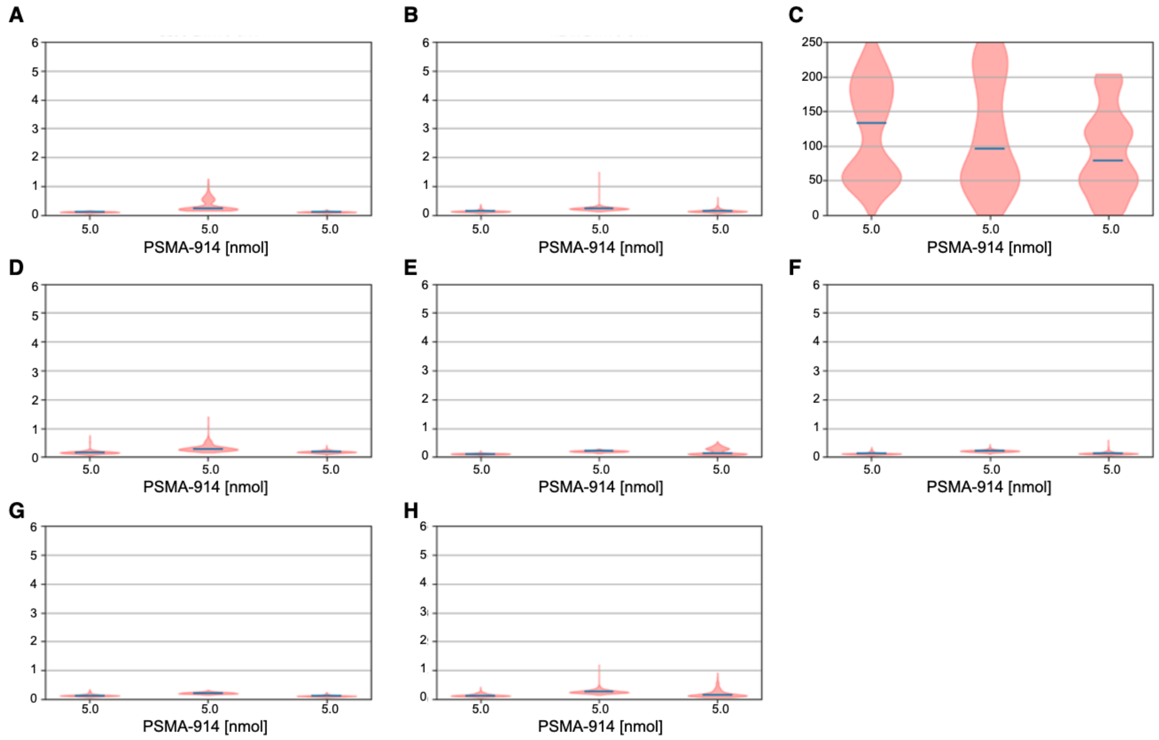


**Supplemental Figure S7. Quantification of *ex vivo* NIR-pCLE imaging of 5 nmol PSMA-914 with the miniprobe GastroFlex$^{TM}$ UHD-C in PC-3 tumor-bearing mice.** Violin plots present the average *ex vivo* signal per pixel, in Analog-to-Digital Units (ADUs), over all recorded frames with a central line indicating the median in (**A**) blood, (**B**) heart, (**C**) kidney, (**D**) liver, (**E**) lung, (**F**) muscle, (**G**) spleen, (**H**) tumor. Information on the sample size (*n*) is given in Supplemental Tables 1 and 3.

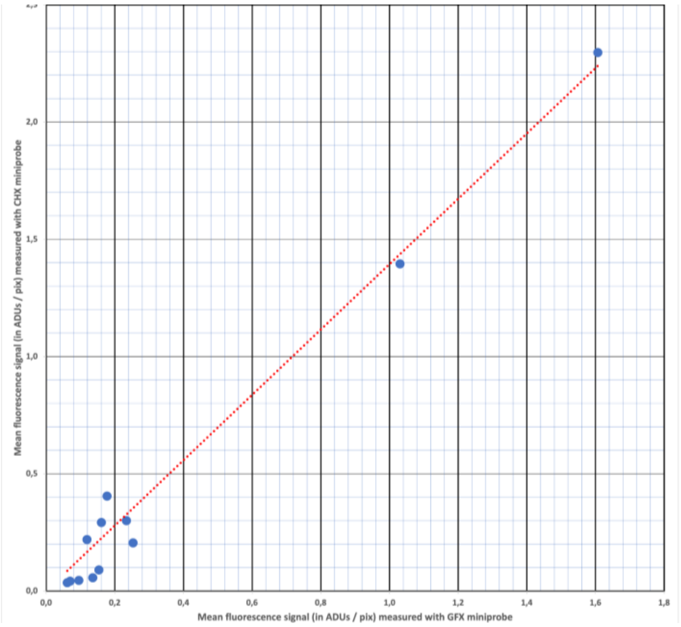


**Supplemental Figure S8. Correlation between the mean *in vivo* and *ex vivo* NIR-pCLE fluorescence signal in LNCaP tumor tissue.** The global average fluorescence intensity per pixel measured *in vivo* with the miniprobe CholangioFlex$^{TM}$-C (CHX) plotted against the global average fluorescence intensity per pixel measured *ex vivo* with the miniprobe GastroFlex$^{TM}$ UHD-C (GFX). The blue data points represent the data corresponding to all analyzed LNCaP tumors. The red line indicates a linear regression with a slope of 1.40 and $R^2$ = 0.98.

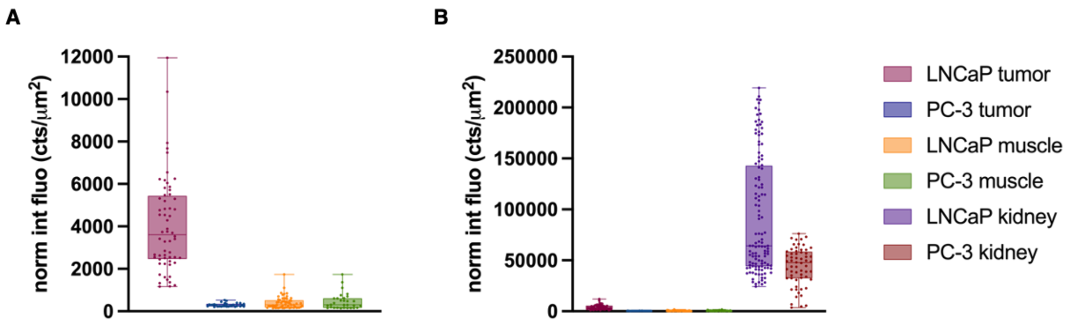


**Supplemental Figure S9. Quantitative comparison of the signal strength of 5 nmol PSMA-914 in confocal imaging of LNCaP/PC-3 tumor, muscle and kidney tissue.** Box plots present the integrated fluorescence intensity per area in confocal images acquired ex vivo with (**A**) LNCaP/PC-3 tumor and muscle tissue cryosections of LNCaP and PC-3 xenograft mice 2 h p.i. of 5 nmol PSMA-914, with a central line indication the median. (**B**) Same as (A), but ordinate scale adapted to also include the data of kidney tissue cryosections. Information on the sample size (*n*) is given in the Supplemental Table 7. Information on significance testing (p-values) is given in Supplemental Table 10.

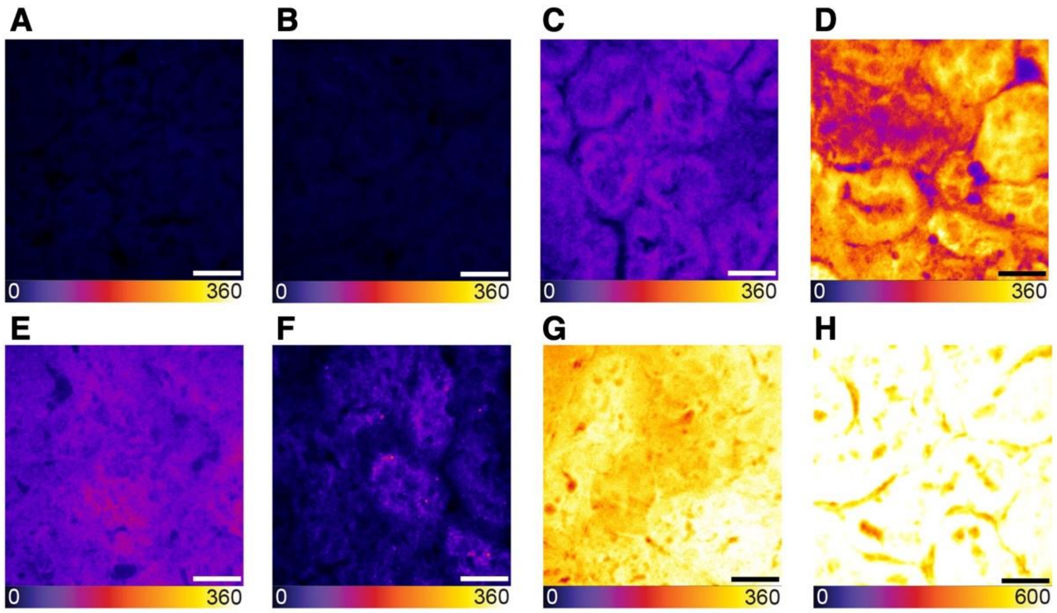


**Supplemental Figure S10. Dose dependent PSMA-914 fluorescence signal in kidney tissue as visualized by confocal microscopy.** Exemplary confocal images of kidney tissue cryosections from LNCaP tumor-bearing mice 2 h p.i. of 0 (**A**)/0.5 (**B**)/1 (**C**)/2 (**D**)/3 (**E**)/4 (**F**)/5 (**G**)/10 (**H**) nmol PSMA-914 (scale bar 20 μm). Raw fluorescence data are shown, color-coded by photon counts per pixel.

# Supplemental Tables

**Supplemental Table S1.** Number of frames collected *ex vivo* with GastroFlex™ UHD-C in organs and tumor of LNCaP-bearing BALB/c nu/nu mice 2h p.i. of PSMA-914.

| Dosing [nmol] | 0.0 | 0.0 | 0.0 | 0.5 | 1.0 | 2.0 | 3.0 | 4.0 | 5.0 | 5.0 | 5.0 | 5.0 | 5.0 | 10.0 |
|---|---|---|---|---|---|---|---|---|---|---|---|---|---|---|
| **Blood** | 334 | 254 | 498 | 340 | 335 | 335 | 333 | 334 | 331 | 333 | 333 | 335 | 350 | 334 |
| **Heart** | 333 | 249 | 496 | 681 | 335 | 334 | 333 | 354 | 395 | 666 | 672 | 675 | 667 | 522 |
| **Kidney** | 663 | 497 | 996 | 2082 | 1041 | 1279 | 667 | 667 | 679 | 665 | 691 | 687 | 669 | 877 |
| **Liver** | 670 | 497 | 994 | 673 | 667 | 667 | 681 | 667 | 733 | 664 | 667 | 668 | 666 | 716 |
| **Lung** | 334 | 249 | 496 | 338 | 333 | 335 | 333 | 341 | 335 | 390 | 342 | 335 | 333 | 337 |
| **Muscle** | 666 | 512 | 996 | 605 | 669 | 664 | 664 | 666 | 736 | 669 | 667 | 671 | 667 | 665 |
| **Spleen** | 474 | 511 | 996 | 675 | 462 | 247 | 663 | 667 | 245 | 665 | 666 | 675 | 668 | 688 |
| **Tumor** | 688 | 498 | 992 | 1582 | 1472 | 1353 | 881 | 667 | 1123 | 1128 | 670 | 700 | 679 | 1071 |

**Supplemental Table S2.** Number of frames collected *in vivo* with CholangioFlex™-C in muscle and tumor of LNCaP-bearing BALB/c nu/nu mice 2h p.i. of PSMA-914.

| Dosing [nmol] | 0.0 | 0.0 | 0.0 | 0.5 | 1.0 | 2.0 | 3.0 | 4.0 | 5.0 | 5.0 | 5.0 | 5.0 | 5.0 | 10.0 |
|---|---|---|---|---|---|---|---|---|---|---|---|---|---|---|
| **Muscle** | 933 | 694 | 1384 | NA | 941 | 932 | 929 | 929 | 929 | 927 | 990 | 931 | 928 | 1022 |
| **Tumor** | 930 | 691 | 1382 | 1201 | 1396 | 1414 | 928 | 931 | 1468 | 925 | 1264 | 1176 | 930 | 963 |

**Supplemental Table S3.** Number of frames collected *ex vivo* with GastroFlex™ UHD-C and *in vivo* with CholangioFlex™-C in organs and tumor of PC-3-bearing BALB/c nu/nu mice 2h p.i. of PSMA-914.

| | ***Ex vivo*, GastroFlex™ UHD-C** | | | ***In vivo*, CholangioFlex™-C** | | |
|---|---|---|---|---|---|---|
| **Dosing [nmol]** | **5.0** | **5.0** | **5.0** | **5.0** | **5.0** | **5.0** |
| **Blood** | 332 | 332 | 333 | NA | NA | NA |
| **Heart** | 666 | 666 | 667 | NA | NA | NA |
| **Kidney** | 667 | 694 | 711 | NA | NA | NA |
| **Liver** | 668 | 682 | 668 | NA | NA | NA |
| **Lung** | 334 | 334 | 331 | NA | NA | NA |
| **Muscle** | 666 | 665 | 668 | 928 | 928 | 929 |
| **Spleen** | 673 | 666 | 667 | NA | NA | NA |
| **Tumor** | 668 | 668 | 669 | 1422 | 951 | 930 |

**Supplemental Table S4.** Total number of frames collected *ex vivo* with GastroFlex$^{TM}$ UHD-C and *in vivo* with CholangioFlex$^{TM}$-C in organs and tumors of LNCaP- and PC-3-bearing BALB/c nu/nu mice 2h p.i. of PSMA-914.

| Total | Probe | LNCaP xenograft | PC-3 xenograft |
|---|---|---|---|
| *Ex vivo* | GastroFlex$^{TM}$ UHD-C | 69 436 | 14 095 |
| | CholangioFlex$^{TM}$-C | 1 295 | NA |
| *In vivo* | GastroFlex$^{TM}$ UHD-C | 455 | NA |
| | CholangioFlex$^{TM}$-C | 28 068 | 6 088 |

**Supplemental Table S5.** Tumor-to-muscle ratio of the median *in vivo* pCLE fluorescence signal detected in tumor and muscle tissue of LNCaP- bearing BALB/c nu/nu mice 2h p.i. of PSMA-914. Probe: CholangioFlex$^{TM}$-C.

| Dosing [nmol] | 0.0 | 0.0 | 0.0 | 0.5 | 1.0 | 2.0 | 3.0 | 5.0 | 5.0 | 5.0 | 10.0 |
|---|---|---|---|---|---|---|---|---|---|---|---|
| Tumor | 1.04 | 1.22 | 0.99 | - | 1.25 | 8.71 | 4.69 | 34.72 | 57.77 | 48.41 | 22.14 |

**Supplemental Table S6.** Organ-to-muscle ratio of the median *ex vivo* pCLE fluorescence signal detected in organ and muscle tissue of LNCaP- bearing BALB/c nu/nu mice 2h p.i. of PSMA-914. Probe: GastroFlex$^{TM}$ UHD-C

| Dosing [nmol] | 0.0 | 0.0 | 0.0 | 0.5 | 1.0 | 2.0 | 3.0 | 5.0 | 5.0 | 5.0 | 10.0 |
|---|---|---|---|---|---|---|---|---|---|---|---|
| Blood | 0.96 | 1.09 | 0.98 | 1.01 | 1.15 | 0.95 | 0.85 | 0.54 | 1.35 | 1.01 | 0.90 |
| Lung | 0.91 | 0.91 | 0.97 | 1.19 | 0.98 | 1.01 | 0.85 | 0.59 | 1.00 | 0.85 | 0.98 |
| Spleen | 1.00 | 0.99 | 0.97 | 1.08 | 1.06 | 0.97 | 0.93 | 0.61 | 1.18 | 1.27 | 1.66 |
| Heart | 1.11 | 0.89 | 0.47 | 1.00 | 1.19 | 1.06 | 1.01 | 0.89 | 1.80 | 2.19 | 2.07 |
| Liver | 1.01 | 0.98 | 0.97 | 1.00 | 1.15 | 1.09 | 0.97 | 1.40 | 1.57 | 3.88 | 8.31 |
| Tumor | 0.96 | 1.03 | 1.01 | 1.91 | 1.64 | 3.15 | 1.33 | 8.31 | 24.51 | 24.93 | 28.98 |

**Supplemental Table S7.** Integrated fluorescence intensity per area as detected with confocal imaging of different PSMA-914 doses in tumor and organ tissue cryosections of LNCaP-bearing BALB/c nu/nu mice 2 h p.i.*.

| PSMA-914 [nmol] | 0.0 | 0.5 | 1.0 | 2.0 | 3.0 | 4.0 | 5.0 | 10.0 |
|---|---|---|---|---|---|---|---|---|
| Tumor | 1375 ± 410 | 1257 ± 373 | 1990 ± 939 | 2575 ± 312 | 2332 ± 511 | 3762 ± 825 | 4042 ± 2204 | 3900 ± 962 |
| No. of frames | 78 | 17 | 10 | 10 | 10 | 10 | 60 | 10 |
| Muscle | 250 ± 76 | 227 ± 25 | 481 ± 229 | 800 ± 178 | 811 ± 175 | 156 ± 22 | 437 ± 275 | 1032 ± 374 |
| No. of frames | 30 | 10 | 10 | 10 | 10 | 10 | 60 | 10 |
| Kidney | 2283 ± 853 | 2480 ± 1262 | 26192 ± 18773 | 118238 ± 20919 | 15201 ± 7739 | 9509 ± 2659 | 93012 ± 57293 | 205347 ± 31452 |
| No. of frames | 50 | 20 | 20 | 10 | 10 | 20 | 120 | 20 |

***** Data are expressed as norm int mean ± SD (counts/$\mu m^2$).

**Supplemental Table S8**. P-values of the quantitative confocal image analysis of different doses of PSMA-914 in tumor tissue cryosections of LNCaP tumor-bearing BALB/c nu/nu mice 2 h p.i.. p<0.05 is considered statistically significant.

| **PSMA-914 [nmol]** | 0 | 0.5 | 1 | 2 | 3 | 4 | 5 | 10 |
|---|---|---|---|---|---|---|---|---|
| 0 | 1.000 | 0.282 | 0.000 | 0.000 | 0.000 | 0.000 | 0.000 | 0.000 |
| 0.5 | 0.282 | 1.000 | 0.008 | 0.000 | 0.000 | 0.000 | 0.000 | 0.000 |
| 1 | 0.000 | 0.008 | 1.000 | 0.080 | 0.332 | 0.000 | 0.006 | 0.000 |
| 2 | 0.000 | 0.000 | 0.080 | 1.000 | 0.239 | 0.001 | 0.042 | 0.001 |
| 3 | 0.000 | 0.000 | 0.332 | 0.239 | 1.000 | 0.000 | 0.019 | 0.000 |
| 4 | 0.000 | 0.000 | 0.000 | 0.001 | 0.000 | 1.000 | 0.697 | 0.747 |
| 5 | 0.000 | 0.000 | 0.006 | 0.042 | 0.019 | 0.697 | 1.000 | 0.844 |
| 10 | 0.000 | 0.000 | 0.000 | 0.001 | 0.000 | 0.747 | 0.844 | 1.000 |

**Supplemental Table S9**. P-values of the quantitative confocal image analysis of different doses of PSMA-914 in muscle tissue cryosections of LNCaP tumor-bearing BALB/c nu/nu mice 2 h p.i.. p<0.05 is considered statistically significant.

| **PSMA-914 [nmol]** | 0 | 0.5 | 1 | 2 | 3 | 4 | 5 | 10 |
|---|---|---|---|---|---|---|---|---|
| 0 | 1.000 | 0.362 | 0.000 | 0.000 | 0.000 | 0.001 | 0.001 | 0.000 |
| 0.5 | 0.362 | 1.000 | 0.004 | 0.000 | 0.000 | 0.000 | 0.020 | 0.000 |
| 1 | 0.000 | 0.004 | 1.000 | 0.004 | 0.003 | 0.000 | 0.634 | 0.001 |
| 2 | 0.000 | 0.000 | 0.004 | 1.000 | 0.891 | 0.000 | 0.000 | 0.110 |
| 3 | 0.000 | 0.000 | 0.003 | 0.891 | 1.000 | 0.000 | 0.000 | 0.126 |
| 4 | 0.001 | 0.000 | 0.000 | 0.000 | 0.000 | 1.000 | 0.002 | 0.000 |
| 5 | 0.001 | 0.020 | 0.634 | 0.000 | 0.000 | 0.002 | 1.000 | 0.000 |
| 10 | 0.000 | 0.000 | 0.001 | 0.110 | 0.126 | 0.000 | 0.000 | 1.000 |

**Supplemental Table S10.** Tumor-to-muscle ratios of the mean integrated fluorescence intensity per area of confocal imaging of different PSMA-914 doses in tumor and muscle tissue cryosections of LNCaP- and PC-3-bearing BALB/c nu/nu mice 2 h p.i.*.

| **PSMA-914 [nmol]** | **0.0** | **0.5** | **1.0** | **2.0** | **3.0** | **4.0** | **5.0** | **10.0** |
|---|---|---|---|---|---|---|---|---|
| **LNCaP** | 5.2 ± 0.8 | 5.5 ± 1.8 | 4.1 ± 2.7 | 3.2 ± 0.8 | 2.9 ± 0.9 | 24.1 ± 6.2 | 9.3 ± 2.0 | 3.8 ± 1.7 |
| **PC-3** | - | - | - | - | - | - | 0.6 ± 0.2 | - |

***** SD was calculated by Gaussian error propagation.

**Supplemental Table S11.** P-values of the quantitative confocal image analysis of 5 nmol PSMA-914 in tumor, muscle and kidney tissue cryosections of LNCaP- and PC-3 tumor-bearing BALB/c nu/nu mice 2 h p.i., $p<0.05$ is considered statistically significant.

| | LNCaP Tumor | PC-3 Tumor | LNCaP Muscle | PC-3 Muscle | LNCaP Kidney | PC-3 Kidney |
|---|---|---|---|---|---|---|
| **LNCaP Tumor** | 1.000 | 0.000 | 0.000 | 0.000 | 0.000 | 0.000 |
| **PC-3 Tumor** | 0.000 | 1.000 | 0.009 | 0.024 | 0.000 | 0.000 |
| **LNCaP Muscle** | 0.000 | 0.009 | 1.000 | 0.696 | 0.000 | 0.000 |
| **PC-3 Muscle** | 0.000 | 0.024 | 0.696 | 1.000 | 0.000 | 0.000 |
| **LNCaP Kidney** | 0.000 | 0.000 | 0.000 | 0.000 | 1.000 | 0.00 |
| **PC-3 Kidney** | 0.000 | 0.000 | 0.000 | 0.000 | 0.000 | 1.000 |

**Supplemental Table S12.** Mean SUV values of PET/MR imaging of 5 nmol $^{68}$Ga-labeled PSMA-914 in LNCaP- and PC-3 tumor-bearing BALB/c nu/nu mice 2 h p.i.*.

| | **LNCaP** | **PC-3** |
|---|---|---|
| **Heart** | 0.08 ± 0.00 | 0.05 ± 0.03 |
| **Spleen** | 0.37 ± 0.18 | 0.15 ± 0.09 |
| **Liver** | 0.42 ± 0.21 | 0.14 ± 0.07 |
| **Kidney** | 4.29 ± 0.93 | 2.99 ±1.48 |
| **Bladder** | 4.32 ± 1.75 | 2.63 ± 0.36 |
| **Muscle** | 0.07 ± 0.00 | 0.04 ± 0.02 |
| **Tumor** | 0.29 ± 0.03 | 0.06 ± 0.04 |

***** Data are expressed as mean ± SD (n=3).

**Supplemental Table S13.** Tumor-to-muscle ratio of the median *in vivo* pCLE fluorescence signal detected in tumor and muscle tissue of PC-3-bearing BALB/c nu/nu mice 2h p.i. of PSMA-914. Probe: CholangioFlex$^{TM}$-C.

| **Dosing [nmol]** | **0.0** | **0.0** | **0.0** |
|---|---|---|---|
| **Tumor** | 3.31 | 1.63 | 0.99 |

**Supplemental Table S14.** Organ-to-muscle ratio of the median *ex vivo* pCLE fluorescence signal detected in organ and muscle tissue of PC-3- bearing BALB/c nu/nu mice 2h p.i. of PSMA-914. Probe: GastroFlex™ UHD-C.

| Dosing [nmol] | 5.0 | 5.0 | 5.0 |
|---|---|---|---|
| **Blood** | 0.81 | 1.09 | 0.76 |
| **Lung** | 0.91 | 0.97 | 1.14 |
| **Spleen** | 1.17 | 1.07 | 0.85 |
| **Heart** | 1.21 | 1.27 | 1.18 |
| **Liver** | 1.64 | 1.70 | 1.75 |
| **Tumor** | 1.20 | 1.34 | 1.38 |

**Supplemental Table S15.** Tumor-to-muscle ratio of the median *ex vivo* pCLE fluorescence signal in tumor and in muscle tissue of LNCaP- and PC-3-bearing BALB/c nu/nu mice 2h p.i. of 0 or 5 nmol PSMA-914*.

| Dosing [nmol] | 0.0 (LNCaP) | 5.0 (LNCaP) | 5.0 (PC-3) |
|---|---|---|---|
| **CholangioFlex™-C** | 1.08 ± 0.10 | 46.97 ± 9.47 | 1.98 ± 0.98 |
| **GastroFlex™ UHD-C** | 1.00 ± 0.03 | 19.25 ± 7.74 | 1.31 ± 0.08 |

***** Data are expressed as mean ± SD (n=3).

**Supplemental Table S16.** Integrated fluorescence intensity per area of confocal imaging of 5 nmol PSMA-914 in organ cryosections of PC-3-bearing BALB/c nu/nu mice 2 h p.i.*.

| Dosing [nmol] | 5.0 |
|---|---|
| **Tumor** | 298 ± 83 |
| **No. of frames** | 30 |
| **Muscle** | 464 ± 378 |
| **No. of frames** | 30 |
| **Kidney** | 43884 ± 18538 |
| **No. of frames** | 60 |

***** Data are expressed as norm int mean ± SD (counts/$\mu m^2$).

**Supplemental Table S17.** P-values of quantitative confocal image analysis of 5 nmol PSMA-914 in PC-3 tumor bearing BALB/c nu/nu mice 2 h p.i.. p<0.05 is considered as statistically significant.

| | Tumor | Muscle | Kidney |
|---|---|---|---|
| **Tumor** | 1.000 | 0.024 | 0.000 |
| **Muscle** | 0.024 | 1.000 | 0.000 |
| **Kidney** | 0.000 | 0.000 | 1.000 |

# References

**1.** Gorlitz FH, P.; Falk, H.J.; Kastrup, L.; Engelhardt, J.; Hell, S.W. A STED Microscope Designed for Routine Biomedical Applications. *Progress in Electromagnetics Research-Pier.* 2014;147:57-68.
